# Intra-database validation of case-identifying algorithms using reconstituted electronic health records from healthcare claims data

**DOI:** 10.1186/s12874-021-01285-y

**Published:** 2021-05-01

**Authors:** Nicolas H. Thurin, Pauline Bosco-Levy, Patrick Blin, Magali Rouyer, Jérémy Jové, Stéphanie Lamarque, Séverine Lignot, Régis Lassalle, Abdelilah Abouelfath, Emmanuelle Bignon, Pauline Diez, Marine Gross-Goupil, Michel Soulié, Mathieu Roumiguié, Sylvestre Le Moulec, Marc Debouverie, Bruno Brochet, Francis Guillemin, Céline Louapre, Elisabeth Maillart, Olivier Heinzlef, Nicholas Moore, Cécile Droz-Perroteau

**Affiliations:** 1 INSERM CIC-P1401, Bordeaux PharmacoEpi, Univ. Bordeaux, Bordeaux, France; 2 Department of Medical Oncology, Hôpital Saint André, CHU de Bordeaux, Bordeaux, France; 3 Department of Urology, University Hospital of Rangueil, CHU de Toulouse, Toulouse, France; 4 Department of Oncology, Clinique Marzet, Pau, France; 5 Department of Neurology, CHRU de Nancy, Nancy, France; 6 Université de Lorraine, EA 4360 APEMAC, Nancy, France; 7 CRC SEP, Neurology Department, CHU de Bordeaux, Bordeaux, France; 8 INSERM U1215, Neurocentre Magendie, Univ. Bordeaux, Bordeaux, France; 9 INSERM CIC 1433 Epidémiologie Clinique, CHRU de Nancy, Nancy, France; 10 Sorbonne Université, Institut du cerveau, ICM, Hôpital de la Pitié Salpêtrière, INSERM UMR S 1127, CNRS UMR 7225, Paris, France; 11 Neurology Department, Hôpital de la Pitié Salpêtrière, APHP, Paris, France; 12 Department of Neurology, Hôpital CHI de Poissy/Saint-Germain-en-Laye, Paris, France

**Keywords:** Validation study, Case-identifying algorithm, Claims database, Reconstituted electronic health record, Multiple sclerosis, Prostate Cancer, Positive predictive value, Negative predictive value

## Abstract

**Background:**

Diagnosis performances of case-identifying algorithms developed in healthcare database are usually assessed by comparing identified cases with an external data source. When this is not feasible, intra-database validation can present an appropriate alternative.

**Objectives:**

To illustrate through two practical examples how to perform intra-database validations of case-identifying algorithms using reconstituted Electronic Health Records (rEHRs).

**Methods:**

Patients with 1) multiple sclerosis (MS) relapses and 2) metastatic castration-resistant prostate cancer (mCRPC) were identified in the French nationwide healthcare database (SNDS) using two case-identifying algorithms. A validation study was then conducted to estimate diagnostic performances of these algorithms through the calculation of their positive predictive value (PPV) and negative predictive value (NPV). To that end, anonymized rEHRs were generated based on the overall information captured in the SNDS over time (e.g. procedure, hospital stays, drug dispensing, medical visits) for a random selection of patients identified as cases or non-cases according to the predefined algorithms. For each disease, an independent validation committee reviewed the rEHRs of 100 cases and 100 non-cases in order to adjudicate on the status of the selected patients (true case/ true non-case), blinded with respect to the result of the corresponding algorithm.

**Results:**

Algorithm for relapses identification in MS showed a 95% PPV and 100% NPV. Algorithm for mCRPC identification showed a 97% PPV and 99% NPV.

**Conclusion:**

The use of rEHRs to conduct an intra-database validation appears to be a valuable tool to estimate the performances of a case-identifying algorithm and assess its validity, in the absence of alternative.

**Supplementary Information:**

The online version contains supplementary material available at 10.1186/s12874-021-01285-y.

## Background

For the last two decades, the use of healthcare databases has considerably increased in health research field [1]. This trend is fueled by the growing recognition that randomized clinical trials, while essential, are not the unique and exhaustive answer to therapeutic efficacy and safety issues. The wealth of information that healthcare databases contain, made them robust tools for many epidemiology-related fields of research, especially in pharmacoepidemiology, where epidemiologic approaches are applied to well-defined and/or large population to assess the use and the effects of drugs in real-world practice [2, 3]. The extensive amount of data collected prospectively and systematically in prolonged period of time, mainly for billing purposes, enables the assessment of infrequent or delayed adverse events as well as therapeutic long-term effectiveness, which is complex to evaluate in classical randomized trials, field cohort or registry [4]. However, the use of secondary data collected for other purposes than epidemiologic research is not devoid of significant limitations [5, 6]. Data quality is a major issue that may impact case identification by inducing a selection or misclassification bias. In studies conducted on healthcare databases, the population or the health outcome of interest is generally identified using in- and/or out-patient diagnosis codes. To enhance accuracy, algorithms including multiple elements specifically related to the studied medical condition (e.g. medical procedures, drug dispensing, laboratory test or radiological exam), in addition to the diagnosis code, may also be developed and implemented [7]. Whatever the approach used, the coding quality may be nuanced in terms of how codes are applied, or how physicians' records are interpreted by medical coders. The financial pressure induced by activity based payment may also lead to encourage the income-maximizing coding of diagnoses and procedures in hospitals at the expense of clinical accuracy [8], although more and more quality audits are carried out to improve coding reliability [9–11]. The validity of algorithm used to identify health outcome in administrative and claims data has always been a matter of concern for researchers, especially in a context of active surveillance and assessment of marketed medical products [12, 13]. Several different types of validation studies may be conducted to assess the fidelity of the codes or algorithms used for cases identification. In all of them, cases identified by the algorithm are compared with a presumably more reliable external diagnostic source or gold standard [7, 14]. These gold standards are most of the time the information that have originated the records in the database (e.g. medical charts or registries) and which contain measure of the disease status based on clinical, biological and/or imaging criteria. The performance of a case-identifying code or algorithm is commonly reported in terms of positive predictive value, sensitivity and specificity. Although necessary, these validation studies are time-consuming and require significant resources and expertise to review diagnoses of clinical data sources. Setting up such a process is also not always possible since the access to the original data source is often complicated or even impossible because of technical or legal issues.

Healthcare databases, are constantly updated with all patient healthcare encounters – medical visits and procedures, laboratory tests or medical imaging, drugs dispensing, hospital stays, etc. – over a considered period of time, or sometimes a lifetime. They may, by their richness and their depth, contain information not available in medical charts. Hence, they may provide a holistic overview of the patient journeys in real-life settings. These longitudinal patient records can be seen as reconstituted Electronic Health Records (rEHRs) and so constitute a valuable alternative to medical charts in validation studies of case-identifying algorithms.

The objective of this paper is to illustrate through two examples of validation studies conducted in the French nationwide healthcare database, the *Système National des Données de Santé* (SNDS) [15], how to perform intra-database validations of case-identifying algorithm using anonymized rEHRs.

## Methods

### Data source

Two validation studies were conducted using data from the SNDS, which currently covers more than 99% of the French population from birth (or immigration) to death (or emigration), even if a subject moves, changes occupation or retires [15, 16]. Using a unique pseudonymized identifier, the SNDS merges all reimbursed outpatient claims from all French healthcare insurance schemes with hospital-discharge summaries from public and private hospitals, and the national death registry. As a consequence, the SNDS contains information on all reimbursed medical and paramedical encounters. For each expenditure, the prescriber and caregiver specialties as well as the corresponding date are provided. The exact quantity of drug dispensed and reimbursed can be identified at the product level with the exact form and dosage. Performed laboratory tests and procedures are available but without results. Registration for Long Term Disease (LTD) – status that ensures a full coverage for all related medical expenses – hospital discharge diagnosis and cause of death are defined using codes from the International Classification of Diseases, 10th revision (ICD-10).

### General method

In the frame of different projects approved by the French regulatory authorities (*Comité d’Expertise pour les Recherches, les Etudes et les Evaluations dans le domaine de la Santé,* CEREES and *Commission Nationale Informatique & Libertés,* CNIL), two algorithms were developed in the SNDS in collaboration with clinical experts of the field to identify: 1) multiple sclerosis (MS) relapses and 2) metastatic castration-resistant prostate cancer (mCRPC). The same methodology for intra-database validation was then applied to each of them in order to ascertain that patients identified as cases or non-cases by the algorithm were respectively true cases and true non-cases.

**Fig. 1:**
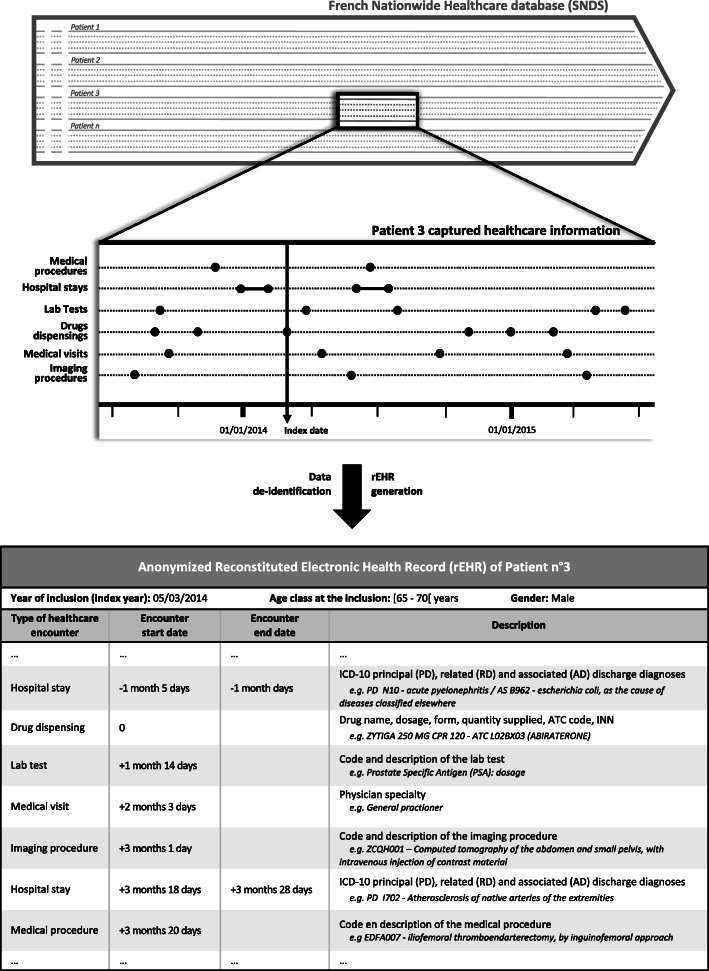
Generation of an anonymized reconstituted Electronic Health Record (rEHR) from data of the French Nationwide Healthcare database (SNDS)

In a first step, anonymized longitudinal rEHRs were generated based on SNDS data for a random selection of 100 patients identified by the algorithm as cases and 100 patients identified by the algorithm as non-cases (Fig. 1). To ensure that individual data contained in these rEHRs did not lead to patient re-identification, new patient identifiers were assigned, calendar dates were replaced by the delay elapsed since inclusion, location details were deleted and only age classes were displayed. In a second step, a validation committee consisting of medical experts of the field, proceeded to a double review of the rEHRs in order to adjudicate on the true case or true non-case status of the selected patients, blinded with respect to algorithm results. In case of discrepancy, all committee members discussed to reach a consensus. In a final step, experts conclusions were compared with algorithm results to estimate its diagnostic performance through the positive predictive value (PPV), the negative predictive value (NPV) and their corresponding 95% confidence intervals (95%CI). The formulae for PPV and NPV were:

$$ PPV=\frac{TP}{TP+ FP} $$

$$ NPV=\frac{TN}{TN+ FN} $$

$$ {\left[95\% CI\right]}_{PPV}= PPV\pm {z}_{1-\frac{\alpha }{2}}\ast \sqrt{\frac{PPV\left(1- PPV\right)}{n_{positive}}} $$

$$ {\left[95\% CI\right]}_{NPV}= NPV\pm {z}_{1-\frac{\alpha }{2}}\ast \sqrt{\frac{NPV\left(1- NPV\right)}{n_{negative}}} $$

where TP and FP are respectively true and false positives, and TN and FN true and false negatives. Corresponding formula for 95%CIs were:

where n_positive_ and n_negative_ are respectively the number of algorithm-based positive and negative assessed cases, and z_(1-α/2)_ the z-value for standard normal distribution with left-tail probability (1-α/2). Here z_(1-α/2)_ = 1.96 for a type I error α = 0.05.

The limitation of the number of rEHRs to be assessed per group to 100 allowed to estimate PPV and NPV with a margin of error < 10% for values above 50%, and made the adjudication of cases possible by experts in less than 48 h.

Following the validation study, experts’ inputs were used to adjust algorithm settings and further improve its discriminatory ability. Overall estimated performances indicators were then updated.

### Case examples

#### Relapses identification in Multiple Sclerosis (MS) patients

The algorithm for identifying relapse in MS was initially developed in the EVIDEMS study whose objective was to assess the effectiveness of dimethyl fumarate versus other MS drugs (i.e. teriflunomide, fingolimod or immunomodulatory injectable drugs) on relapses after treatment initiation [17]. The study cohort included all patients identified in the SNDS by a first dispensing of MS drug (i.e. dimethyl fumarate, indicated for MS) between July 2015 and December 2017, with 4.5-year history and 1 to 3.5 years of follow-up. Relapses were identified using an algorithm combining dispensing of high dose corticosteroids (methylprednisolone or betamethasone) and hospital discharge diagnoses related to MS (multiple sclerosis, encephalitis, myelitis, encephalomyelitis, optic neuritis) [17]. A minimum lag of 31 days was required to consider two relapses as independent. Further details about the algorithm are provided in Additional file 1.

#### Patients with metastatic Castration-Resistant Prostate Cancer (mCRPC)

The algorithm for mCRPC patients identification was initially developed in the CAMERRA study whose objectives were to assess mCRPC burden and describe mCRPC-specific treatment lines [18, 19]. The study cohort included all patients with a prostate cancer identified in the SNDS by a specific hospital discharge or LTD diagnosis code or a specific treatment dispensing between January 2009 and December 2014, with 5-year history and 3 years of follow-up. Patients with mCRPC were identified using an algorithm integrating time indicators related to metastases management and castration resistance. Both indicators relied on the detection of specific procedures (e.g. imaging, surgery or radiotherapy), drug dispensing (e.g. androgen deprivation therapy, metastases-targeted treatment, chemotherapy) or specific hospitalizations. A complete description of the algorithm and its validation are available elsewhere [18]. In the CAMERRA validation study, so as to ensure the presence of all categories of non-mCRPC patients, three groups of non-mCRPC patients were identified: 34 with non-metastatic hormone-sensitive prostate cancer, 33 with metastatic hormone-sensitive prostate cancer, and 33 with non-metastatic castration-resistant prostate cancer. A single NPV relying on the overall non-mCRPC population was then estimated for the algorithm by weighting false-negative cases according to the actual distribution of the 3 categories of non-mCRPC patients in the prostate cancer population. In a last stage, PPV, NPV and the observed prevalence of mCRPC among the study population (prostate cancer patients), were used to derived the sensitivity and specificity [20].

## Results

### Diagnostic performance of the MS relapse algorithm

A sample of 200 patients was randomly selected from the initial study population; 100 of them had at least one relapse and 100 did not have any relapse according to the algorithm. The validation committee confirmed 95 patients with relapses (true cases) among the algorithm-identified cases and 96 without relapse among the algorithm-identified non-cases, resulting in a PPV of 95.0% (95%IC = [91; 99]) and a NPV of 96.0% (95%IC = [92; 100]) (Additional file 2, Table A). After the update of algorithm settings based on experts’ conclusions, NPV reached 100.0% (Table 1)

**Table 1:** Positive (PPV) and negative (NPV) predictive values of the final algorithm for the identification of relapse in multiple sclerosis

		Validation committee			
		Relapse +	Relapse -	Total	
**Algorithm**	**Relapse +**	99	5	104	PPV = 95% (95%CI = [91; 99])
	**Relapse -**	0	96	96	NPV = 100%
	**Total**	99	101	200	

### Diagnostic performance of the mCRPC algorithm

A sample of 200 patients was randomly selected from the initial population with prostate cancer; 100 of them were identified as mCRPC and 100 as non-mCRPC according to the algorithm. Experts confirmed 92 of the 100 algorithm-identified mCRPC cases and 93 of the 100 algorithm-identified non-mCRPC cases, resulting in a PPV and NPV of respectively 92.0% (95%CI = [87; 97]) and 99.0% (95%CI = [98; 100]), after weighting according to non-mCRPC cases distribution (Additional file 2, Table B). Following the algorithm adjustment based on expert feedback, PPV reached 97% (95%CI = [93; 100]) (Table 2). Based on an observed proportion mCRPC/prostate cancer of 3.4%, sensitivity and specificity of the final algorithm were respectively estimated at 80 and 100% [18].

**Table 2:** Positive (PPV) and negative (NPV) predictive values of the final algorithm for the identification of metastatic castration-resistant prostate cancer (mCRPC), adapted from Thurin NH, et al. 2020

		Validation committee			
		mCRPC +	mCRPC -	Total	
**Algorithm**	**mCRPC +**	90	3	93	PPV = 97% (95%CI = [93; 100])
	**mCRPC -**	1.23^a^	105.77^a^	107	NPV = 99% (95%CI = [97; 100])
	**Total**	91.23	108.77	200	

^a^After weighting

## Discussion

Based on two practical examples relying on the French nationwide healthcare database, this paper illustrates an innovative method to assess case-identifying algorithms, conducting an intra-database validation study. In both examples, this validation study showed that algorithms had high diagnostic performances, with excellent PPV and NPV. To our knowledge, there are no previous examples of the use of rEHRs to assess the performances of algorithms for case identification, therefore results are difficult to compare with existing data. Because by law, returning to individual medical records from SNDS data is forbidden, most of the currently published French validation studies were limited to the comparison of hospital discharge codes extracted from local hospital databases – before their de-identification and integration to the SNDS – with traditional sources of information such as medical charts or registries, and leading to a PPV varying from 80 to 90% but tending to decrease according to the granularity of the required information [21–27].

In the present case, intra-database validation provides the opportunity to assess algorithms that rely on multiple elements from SNDS, enabling to improve discriminatory abilities compared to single identification criterion or to overcome the absence of a direct-identifying diagnostic code [28]. Experts of the validation committee reported that rEHRs proceeding from SNDS data were on certain points more informative than the fragmented information usually enclosed in traditional medical charts, and contained a high level of details as well as an accurate chronology regarding patient journeys, which generally made the adjudication of the cases non-ambiguous. Clinicians insights also allowed to refine the algorithm, adjusting its settings to further improve its performances. This suggests that SNDS data are comprehensive enough to develop a complex algorithm and to validate it.

As the SNDS captures the exhaustivity of reimbursed healthcare encounters in France, the absence from a rEHR of an element that is supposed to be captured by the database is synonymous with the absence of the corresponding healthcare encounter in real life, meaning that this element will not be present in the patient’s medical chart either. In the event that a pre-specified sequence of cares that is essential for the case identification is not captured by the database although the disease or outcome is really present, only the number of false negatives detected by the algorithm will be impacted. As a consequence, only the algorithm sensitivity will be affected but the PPV, which represents the reliable identification of actual cases, will remain unchanged.

Validation studies based on medical charts review stay the best way to evaluate claims database algorithms. However, it requires a lot of human time and reliable significant funding, which are often missing, to be able most often to estimate only the PPV. Wherever feasible, validation studies relying on linkage between administrative databases and medical registries or electronic medical record databases are a good alternative, but they are rarely fully representative of the whole database population, and remain quite long and expensive. Conversely, rEHR review offer a time- and cost-efficient way to conduct validation studies, using the data source accessed by the algorithm. Files to review are standardized and structured, allowing the assessment of hundreds of cases in a limited time: 50 cases per expert-day in the two presented examples. This means that 2 days with 2 experts are sufficient to conduct a double review of 100 cases, with a precision ≤7% for a PPV or NPV ≥ 80%, and ≤ 6% for a PPV or NPV ≥ 90%. By increasing the number of cases to review to 200, precisions improved to respectively ≤6% and ≤ 4%. Moreover, as both cases and non-cases are accessible, this approach enables the calculation of other indicators than PPV (e.g. NPV, sensitivity, specificity), with a full representativeness of the population covered by the database.

We acknowledge that validating an algorithm in the same database that was used to develop it may be questionable. The suitability of using an unique data source to generate and evaluate a hypothesis has been previously discussed in the scientific literature, even if the scope was slightly different [29–32]. Walker AM., and Wang SV. and colleagues argued that for such an approach to be considered valid “test data need to be independent of hypothesis-generating data” [29, 30]. Though it is consensual that re-using data to perform quality check, reevaluate findings and strengthen hypotheses (e.g. sensitivity analyses) in the frame of pharmacoepidemiology studies belongs to good research practice, the fact that they can also be used to validate hypotheses is more challenged, especially because of the potential lack of argument to establish causality [31]. In hypothesis-evaluating treatment effectiveness studies, the reuse of data sources is usually not recommended upon the main argument that it leads to replication rather than confirmation [30, 32]. In the present work the lack of argument to establish causality is not an issue, as we do not seek it; the unique objective is to prove that cases identified by the algorithm are true cases. Moreover, here, the independence is ensured by the unrelated approaches used in the identification and the confirmation of the cases: to classify a case, the algorithm picks up information in the database as previously defined in a statistical analysis plan. When experts do so, they choose the relevant information for themselves in the rEHR. Relying on the same data, the elements considered can be the same (or not), but the approach and the selection process are different, resulting in independent bodies of proof.

Obviously, preference must be given to external data source to conduct validation study, especially those encompassing re-interpretable clinical elements that could lead experts to reconsider the initial diagnosis, even in the absence of details on patient sequence of cares. But when it is not feasible, the re-use of the original data source appears as a valuable alternative. Moreover, it should be borne in mind that the decision whether or not to proceed with intra-database validation for case-identifying algorithm will strongly depend both on the nature of the outcome of interest and on the characteristics of the considered database. Two conditions must be fulfilled to ensure an effective application of the method: 1) the health outcome of interest must be managed by a specific sequence of cares and encounters; 2) the considered healthcare database must capture in an exhaustive way a sufficient number of medical elements in line with the outcome of interest.

Outcome validation should not rely on a unique diagnostic or procedure code but on several tangible elements. As a consequence, intra-database validation should only be considered for health outcomes that are managed in usual clinical practice by a well-defined chronological sequence of cares (procedures, drug dispensing, hospital stays, medical visits, etc.) since diagnostic evidence – such as images or laboratory results – may be absent of the database. The succession of healthcare encounters that individually may be unspecific of the outcome, when taken together, give rise to a specific healthcare pathway. This is particularly true for serious outcomes, mobilizing large healthcare resources. Thus, chronic conditions such as MS (see example 1) or cancer (see example 2), or serious acute outcomes for which the management follows consensual and structured guidelines (e.g. myocardial infarction) [33] seem to be better suited to intra-database validation, compared to non-serious outcomes involving few and unspecific healthcare resources (e.g. acute sore throat) [34], or serious but rare diseases with no clinical practice guidelines [35]. Particular attention must be paid to the clinical guidelines which were ongoing at the time of the study, since they drive patient journeys and thus, experts’ judgment.

Furthermore, in order to ensure that rEHRs provide sufficient and reliable information to enable case adjudication, the underlying healthcare database must capture a sufficient number of medical elements in an exhaustive way over a suitable period of time. Data collected must be, at least, in line with the type of care involved in the management of the outcome of interest (e.g. validation of a myocardial infarction identification requires, as a minimum, in patient data). Ideally, outpatient and inpatient healthcare encounters should be included, and the quality of the captured information regularly assessed. Data completeness, at least over the study period, is mandatory to ensure that the absence of record is synonym of an absence of encounter. The SNDS is particularly well suited to this situation since it fulfills all these requirements: it includes in- and out-patient information of all reimbursed healthcare encounters, most of the time lifelong, and the quality of coding is ensured by regular internal and external audits [9–11].

## Conclusion

Homogeneous healthcare databases such as the SNDS captures healthcare journey of patients lifelong. Although these data cannot replace the anamnesis and the clinical information reported in patient medical charts, this succession of healthcare records appears to be comprehensive enough to generate consistent rEHRs assessable by experts, allowing to conduct validation studies without using external information. It should be made clear that intra-database validation based on rEHRs review does not pretend to replace traditional methods of validation relying on medical charts review. However, as illustrated here through the MS relapse example and the mCRPC example, in the absence of alternative, such method appears to be a valuable tool to estimate the performances of case-identifying algorithms and assess their validity. The development in the coming years of data linkages allowing to gather claims data, registries, electronic health records, etc. [36, 37], will further enrich data available for experts to review in rEHRs, and may blur the line between intra-database validation and external medical chart review.

## Supplementary Information

**Additional file 1: ** Algorithm for the identification of multiple sclerosis relapses.

**Additional file 2: ** Algorithm performance indicators before setting adjustment resulting from expert inputs.

## Data Availability

As per law raw SNDS data cannot be shared. To date, access to SNDS data requires approval from the *Comité Ethique et Scientifique pour les Recherches, les Etudes et les Evaluations dans le domaine de la Santé* (CESREES) in charge of assessing scientific quality of the project, and authorization from the *Commission Nationale de l’Informatique et des Libertés* (CNIL) which is the French data protection authority, and then an agreement with the SNDS data holder (CNAM).

## Abbreviations

CEREES: Comité d’Expertise pour les Recherches, les Etudes et les Evaluations dans le domaine de la Santé
CNIL: Commission Nationale Informatique & Libertés
FN: False negative
FP: False positive
ICD-10: International classification of diseases, 10th revision
LTD: Long term disease
mCRPC: metastatic castration-resistant prostate cancer
MS: Multiple sclerosis
NPV: Negative predictive value
PPV: Positive predictive value
rEHR: reconstituted Electronic Health Record
SNDS: Système National des Données de Santé
TN: True negative
TP: True positive
95%CI: 95% confidence interval
