# Supplementary material for: Intra-database validation of case-identifying algorithms using reconstituted electronic health records from healthcare claims data
Source: BMC Med Res Methodol. 2021 May 1;21:95. doi: 10.1186/s12874-021-01285-y (PMC8088022; doi:10.1186/s12874-021-01285-y)
Supplement: Supplementary file 1 — Additional file 1: Algorithm for the identification of multiple sclerosis relapses. [file 12874_2021_1285_MOESM1_ESM.pdf]

ADDITIONAL FILE 1 – Algorithm for the identification of multiple sclerosis relapses.

Hospital discharge diagnoses codes considered for multiple sclerosis (MS) relapse (International Classification of Diseases, 10th edition [ICD-10] codes)

- Multiple sclerosis (G35)
- Encephalitis, myelitis and encephalomyelitis, unspecified (G04.9)
- Other encephalitis, myelitis and encephalomyelitis (G04.8)
- Optic neuritis (H46)
- Retrobulbar neuritis in diseases classified elsewhere (H48.1)

Corticosteroids considered (Anatomical Therapeutic Chemical [ATC] Classification):

- Methylprednisolone (H02AB04)
- Betamethasone (H02AB01)

Relapses were identified on patients with no hospital stay when dispensing of at least 2g of corticosteroid were observed within 10 days.
Hospital stay of 1 or 2 days for MS had to be followed by respectively at least 2g or 1g of high dose corticosteroid within 10 days to ascertain the relapse diagnosis.
The presence of an ICD10 code Z51.2 for 'other chemotherapy' (*i.e. immunosuppressant)* code associated to ≥2 days of hospitalization within 10 days was also used as indicator.
Finally, hospital stay of 3 to 5 consecutive days, only associated with a MS with no other diagnostic, was also classified as a relapse.
A minimum lag of 31 days was required to consider two relapses as independent.

Note: Hospital stays / hospitalisations encompassed both day hospital stay, and overnight hospital stay.

Reference: Pauline Bosco-Levy, Marc Debouverie, Bruno Brochet et al. Comparative effectiveness of dimethyl fumarate in Multiple Sclerosis, 12 March 2021, PREPRINT (Version 1) available at Research Square [https://doi.org/10.21203/rs.3.rs-321622/v1]
