# Supplementary material for: Intra-database validation of case-identifying algorithms using reconstituted electronic health records from healthcare claims data
Source: BMC Med Res Methodol. 2021 May 1;21:95. doi: 10.1186/s12874-021-01285-y (PMC8088022; doi:10.1186/s12874-021-01285-y)
Supplement: Supplementary file 2 — Additional file 2: Algorithm performance indicators before setting adjustment resulting from expert inputs. [file 12874_2021_1285_MOESM2_ESM.pdf]

ADDITIONAL FILE 2: Algorithm performance indicators before setting adjustment resulting from expert inputs

Table A. Positive (PPV) and negative (NPV) predictive values of the algorithm for the identification of relapse in multiple sclerosis before settings adjustment

		Validation committee		
		**Relapse +**	**Relapse -**	**Total**
**Algo-ithm**	**Relapse +**	95	5	100
	**Relapse -**	4	96	100
	**Total**	99	101	200

PPV = 95% (95%CI = [91; 99])
NPV = 96% (95%CI= [92; 100])

Table B. Positive (PPV) and negative (NPV) predictive values of the final algorithm for the identification of metastatic castration-resistant prostate cancer (mCRPC) before settings adjustment

		Validation committee		
		**mCRPC +**	**mCRPC -**	**Total**
**Algo-ithm**	**mCRPC +**	92	8	100
	**mCRPC -**	0.75*	99.25*	100
	**Total**	92.75	107.25	200

PPV = 92% (95%CI= [87; 97])
NPV = 99% (95%CI= [98; 100])

**After weighting*
